# Interventions for positive parental feeding practices in low-income households: a scoping review

**DOI:** 10.1186/s12966-026-01938-5

**Published:** 2026-06-17

**Authors:** Mohammad Affendy Mhd Akhir, Norhasmah Sulaiman, Nurzalinda Zalbahar, Hanina Hamsan

**Affiliations:** 1 Department of Nutrition, Faculty of Medicine and Health Sciences, Universiti Putra Malaysia, Serdang, 43400 Selangor Malaysia; 2 Department of Social and Development Sciences, Faculty of Human Ecology, Universiti Putra Malaysia, Serdang, 43400 Selangor Malaysia

**Keywords:** Responsive feeding, Parental feeding practices, Low-income households, Parenting interventions, Early childhood nutrition, Household food insecurity

## Abstract

**Background:**

Parental feeding practices play a critical role in shaping children’s dietary behaviours and self-regulation during early childhood. These practices are particularly important in low-income households, where structural constraints, including household food insecurity (HFI), may influence how feeding guidance is implemented and sustained. Although a growing number of interventions aim to promote positive parental feeding practices, there remains limited synthesis of how contemporary programmes are conceptualised, designed, and delivered within low-income contexts, including the extent to which feeding-related components, implementation strategies, and contextual factors such as HFI are addressed.

**Methods:**

A comprehensive literature search was conducted across PubMed, CINAHL, and EMBASE using predefined search terms. The study selection followed the Joanna Briggs Institute methodology for scoping reviews and the PRISMA-ScR guidelines. Eligible studies included intervention programmes targeting parental feeding practices among low-income families with young children.

**Results:**

Sixteen articles representing fifteen unique interventions published between 2015 and 2025 were included. The parenting-focused domain was the most common, followed by digital, multicomponent, and home-based nutrition education and skill-building domains. Across interventions, responsive feeding emerged as the most consistently targeted and improved construct, regardless of delivery mode or setting. Interventions incorporating active skill-building strategies such as coaching, modelling, and guided practice were more consistently associated with changes in parental feeding behaviours than those relying primarily on information provision. Explicit integration of HFI into the intervention design was limited; only a small number of interventions (*n* = 3) measured or directly addressed food insecurity, while others (*n* = 2) addressed related constraints implicitly through food provision, stress reduction, or improvements in home food availability. The effects on child anthropometric outcomes were generally limited and inconsistent.

**Conclusions:**

Contemporary feeding interventions in low-income households demonstrate substantial conceptual convergence around responsive feeding yet vary widely in implementation strategies and attention to structural context. Findings suggest that feeding practices, meal structure, and the home food environment may represent more appropriate intermediate indicators of intervention success than anthropometric outcomes in early childhood. Future interventions should prioritise theory-driven, skill-based designs and more explicitly integrate household food insecurity and related contextual constraints to enhance relevance, equity, and sustainability.

**Trial registration:**

Open Science Framework (OSF), January 12, 2026 (DOI: https://doi.org/10.17605/OSF.IO/RHSBF.

**Supplementary Information:**

The online version contains supplementary material available at https://doi.org/10.1186/s12966-026-01938-5.

## Background

Food parenting practices refer to the strategies caregivers use to structure, guide, and respond to children’s eating [1]. These practices shape children’s eating behaviours from early childhood onwards and play a central role in their emerging self-regulation and dietary preferences [2]. According to Chen et al. [3], positive food parenting practices, such as encouraging autonomy and providing healthy options, are linked to improved dietary outcomes in children, signalling the crucial role that parents or caregivers play as primary agents of socialisation in dietary habits. Positive reinforcing strategies, such as role modelling healthy eating or providing autonomy in food choices, foster healthier eating habits among children [4]. By contrast, coercive practices such as using food as a reward or punishing unhealthy choices can lead to adverse eating behaviours, including emotional eating or a preference for unhealthy foods [5]. A significant finding by Jansen et al. [6] indicates that parents who demonstrate high levels of structure and support at mealtimes create an environment conducive to healthy eating, whereas those exhibiting high levels of restriction may inadvertently promote unhealthy relationships with food. Structure and support refer to parental organisation of the eating environment (e.g., meal routines and availability of healthy foods) while restriction refers to limiting children’s access to specific foods to control intake [7]. As children grow, the consequences of food parenting practices become increasingly evident in their long-term health outcomes. Positive food parenting practices have been associated with a lower risk of obesity and disordered eating later in life [8]. In addition, studies show that negative food parenting practices are associated with higher incidences of malnutrition and psychological issues related to dietary intake, thus establishing a compelling connection between early food practices and adolescent health trajectories [9].

Current research into food parenting practices is expanding, emphasising the need to understand not only the effects of food parenting on child outcomes but also the psychosocial and socio-cultural factors that influence these practices. Studies, including those by Mou et al. [10] and Douglas et al. [11], show that factors like parental mental health, cultural norms, and food security are critical variables affecting food parenting practices and child nutrition. Parental psychosocial well-being significantly impacts their food parenting practices. Jansen et al. [6] found that increased parental stress is often associated with increased use of coercive food practices, such as pressure to eat and emotional eating behaviours. Given the complex interaction between parental mood and food-related practices, it is important to consider psychosocial factors when designing interventions aimed at improving child dietary behaviours. In a longitudinal study, it was shown that parents experiencing higher levels of anxiety tend to use more restrictive feeding practices, affecting their children’s relationship with food [12]. Parents’ own eating behaviours and attitudes also serve as strong predictors for their children’s dietary patterns. Hevesi et al. [13] detail how maternal modelling of positive feeding behaviours can foster healthier child feeding habits, while negative parental feeding behaviours foster maladaptive feeding patterns in their children, emphasising the importance of modelling healthy feeding practices.

Significant variations in food parenting practices also exist across different ethnic and socioeconomic groups. Research suggests that economic instability can force families to adopt less healthy food parenting practices due to limited resources, negatively affecting children’s dietary choices [14]. These disparities highlight a critical direction for future research aimed at understanding how socioeconomic factors intersect with food parenting practices. The outcomes and types of interventions focused on food parenting practices in low-income households remain insufficiently specified. There has not been a study that compiles all forms of intervention programme factors and examines the effects and interactions of each factor. While the scoping review by Baxter et al. [15] provided an important foundation for understanding parental feeding practices in the context of socioeconomic disadvantage, the evidence base has expanded substantially in recent years. Baxter et al. [15] highlighted household food insecurity as a critical contextual factor shaping parental feeding practices, and they noted that many interventions targeting feeding behaviours inadequately addressed the structural conditions under which families make feeding decisions. Building on this work, the present scoping review examines how contemporary interventions implemented in low-income contexts operationalise positive parental feeding practices and the extent to which household food insecurity is explicitly or implicitly considered within the intervention design. The present review builds on this work by incorporating more recent intervention studies published between 2015 and 2025, increasing the total number of interventions synthesised to 15. Also, in contrast to earlier reviews, this study applies a component-based analytical framework to examine how specific intervention strategies are associated with feeding, dietary, behavioural, and anthropometric outcomes. This updated synthesis is necessary to inform the development of future interventions that are responsive to contemporary delivery modes and the complex needs of families experiencing disadvantage. Therefore, the purpose of this scoping review of the literature published in the past decade is to investigate the key conceptual foundations underpinning existing intervention programmes designed to promote positive parental feeding practices within low-income households, with particular attention to the predominant types of interventions and implementation strategies employed. A better understanding of the current interventions will be essential for advancing the field. This knowledge will help identify the strengths and limitations that need to be considered in developing and evaluating future interventions to promote positive parental feeding practices.

## Methods

This review was written based on three (online) databases conducted according to the Joanna Briggs Institute’s guidelines and protocols [16], the PRISMA-ScR checklist [17], and the five research steps proposed by Arksey and O’Malley’s [18] study framework whenever applicable. The review protocol and progress were discussed and recorded during weekly team meetings. The protocol was registered retrospectively on 12 January 2026, in the Open Science Framework (OSF) (doi: https://doi.org/10.17605/OSF.IO/RHSBF).

### Stage 1- Identify the research questions

Scoping review questions:

1. What are the key concepts (focusing on the types of intervention and the most common implementation strategies) applied in intervention programmes to promote positive parental feeding practices among low-income households?
2. What are the reported outcomes of the interventions?
3. To what extent do feeding interventions explicitly or implicitly consider household food insecurity (HFI) in their design or evaluation?

### Stage 2- Identify relevant studies

Between 17 November 2025 and 21 November 2025, a search for related articles using the keywords (“parent feeding” OR “feeding practices” OR “food parenting” OR “feeding style” OR “feeding behavior” OR “feeding behaviour” OR feeding OR nutrition OR diet OR “positive feeding practices” OR “parental feeding practices” OR “nutrition education”) AND (intervention OR program OR programme OR strategy OR education OR training OR module OR promotion OR “parenting program” OR “parenting programme”) AND (“low-income” OR “low income” OR “economically disadvantaged” OR poverty OR disadvantaged OR deprived OR “food insecurity” OR indigenous OR native OR aboriginal OR “first nations”) AND (child OR children OR infant OR preschool OR toddler) was conducted in three databases: PubMed, CINAHL, and EMBASE.

### Stage 3- Study selection

Inclusion criteria for the review were developed using the PCC (Population, Concept, and Context) framework. All records identified from database searches were exported into reference management software and duplicates were removed. The screening process was conducted in two stages. First, titles and abstracts were screened to identify records potentially relevant. Second, the full-text publications of the remaining records were retrieved and assessed against all predefined eligibility criteria. Records included in the review met the inclusion and exclusion criteria.

The following were the inclusion criteria:

1. Intervention studies with a parenting or feeding practice component.
2. Parents or primary caregivers of children aged under five years old (Population).
3. Interventions promoting positive food parenting practices, such as responsive feeding, feeding styles, meal structure, and autonomy support) (Concept).
4. Populations described as low-income, resource-constrained environments, disadvantaged, or food insecure (Context).
5. Articles published in English.
6. Articles published between 2015 and 2025.

Records were excluded if they:

1. Did not involve an intervention study.
2. Did not focus on food parenting practices or parental feeding behaviours.
3. Involved populations outside the defined criteria.
4. Were conference abstracts, editorials, commentaries, or review articles.

Two reviewers (M.A.M.A and N.S) independently screened the articles and the results were compared during a single discussion session. At times, when a discrepancy occurred, a third and fourth reviewer (N.Z and H.H.H) would be requested for consultation. No additional criteria were set for the duration of interventions or intervention comparison. Any published articles that did not meet the above inclusion criteria were excluded from the review.

### Stage 4- Charting the data (data extraction)

Following the research questions, full articles were retrieved from pertinent sources and analysed. Two evaluators (M.A.M.A and N.S) independently reviewed each article and documented intervention characteristics using items from the Template for Intervention Description and Replication (TIDieR) for describing components of intervention including the what, who, how, where, when and how much intervention is delivered to allow future replication or scale-up [19, 20].

### Stage 5- Collating, summarising and reporting results

In the scoping review, it is important to include multiple data to determine the result of the study topic and the manner in which the included data are represented is also important [21]. All the identified articles were collected, examined, and reported based on the following themes: (1) intervention characteristics of included studies according to the TIDieR checklist, (2) intervention domains and implementation strategies, and (3) consideration of HFI in intervention studies. These data are discussed in the results section of this review. Since this study was a review of research articles, neither ethical approval nor individual consent was needed.

## Results

### Intervention characteristics of included studies according to the TIDieR checklist

**Fig. 1:**
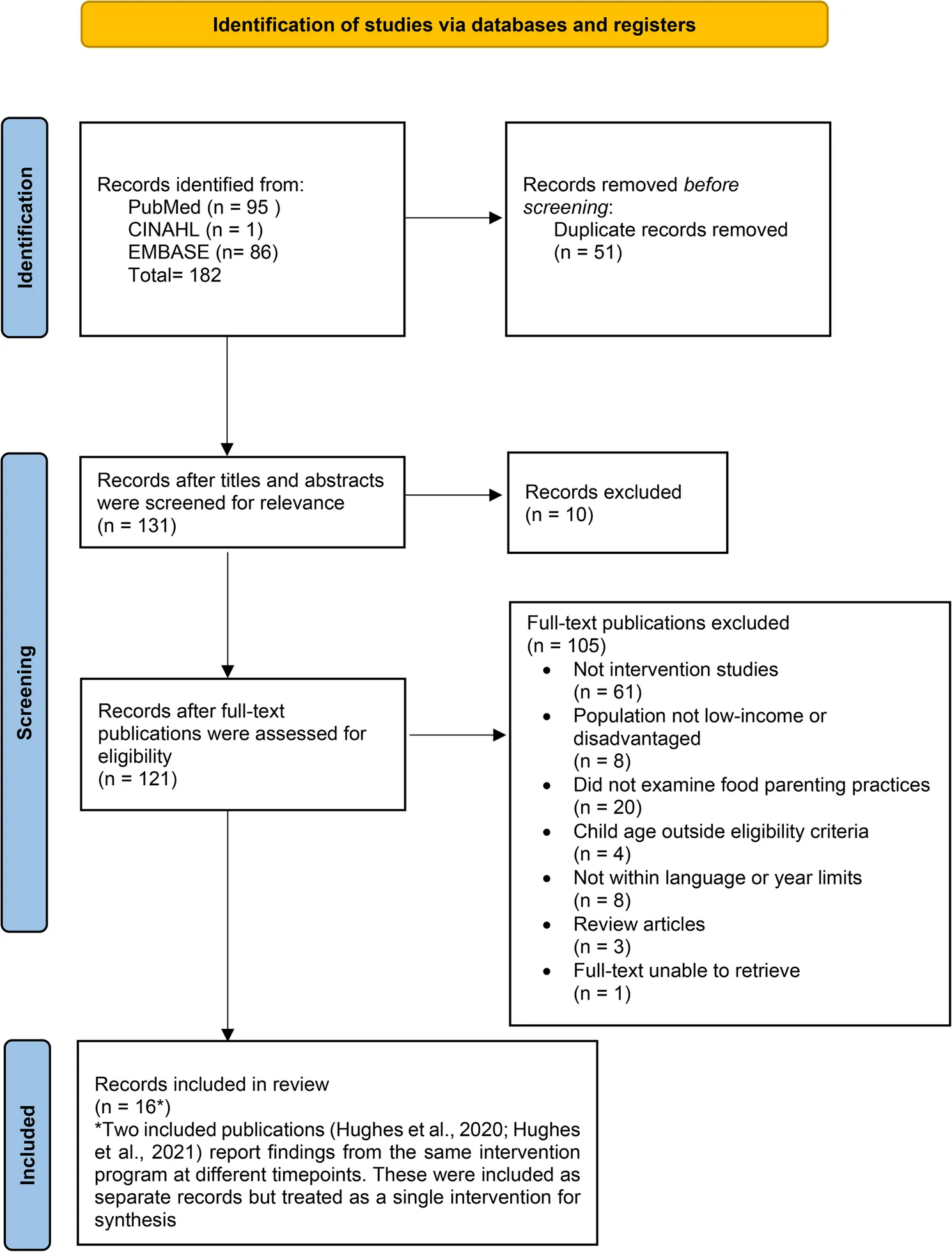
PRISMA flow diagram of study selection process

Following the literature search, a total of 182 records were identified: 95 PubMed, 1 CINAHL, and 86 EMBASE. After removing duplicate records (*n* = 51), 131 records were screened for relevance based on the titles and abstracts. Of these, 10 records were excluded for irrelevance. 104 records did not meet the eligibility criteria and one article was unable to be retrieved for full text, which reduced the total to 16 records [21–36]. Figure 1 displays the PRISMA flow diagram of the study selection process. This comprehensive scoping review largely included previous studies on intervention programmes conducted among low-income, vulnerable, and economically disadvantaged populations that employed positive parental feeding practices. Tables 1 and 2 provide an overview of the characteristics of included studies. The 15 studies included in this review encompassed diverse methodological study designs, with the majority being individually randomised controlled trials (*n* = 8), cluster randomised design (*n* = 1), pragmatic trial (*n* = 1), randomised pilot studies (*n* = 2), secondary analysis of randomised trial (*n* = 1), quasi-experimental pilot study (*n* = 1), and mixed method randomised trial (*n* = 1). Publication dates ranged from 2017 to 2025, with 13 studies published in 2020 or later, reflecting the growing attention to this research area in the past five years. Regarding the sample country, the majority of studies (*n* = 11) were conducted in the United States across multiple geographic regions, including Texas, Washington, New York and other locations. While most interventions were implemented in the United States, no studies were identified from Southeast Asian countries, highlighting a geographical research gap. Sample sizes ranged substantially from 32 participants in the smallest pilot study to 662 participants in a pragmatic trial, with a total sample size of approximately 3,295 participants in these 15 intervention studies.

**Table 1:** Characteristics of studies included in the review (*n* = 15)

Characteristic	Description	Number (*n*)	Percentage (%)
Study design	Individually randomised controlled trials Cluster randomised trial Pragmatic trial Randomised pilot studies Secondary analysis of randomised trial Quasi-experimental pilot study Mixed method randomised trial	8 1 1 2 1 1 1	56.25 6.25 6.25 12.5 6.25 6.25 6.25
Publication year	2025 or later 2024 − 2020 2019 − 2015	3 9 3	18.75 62.50 18.75
Sample country	USA Ethiopia Nigeria Australia	12 1 1 1	81.25 6.25 6.25 6.25
Sample size	1–19 20–39 40–59 60–79 80–99 100–999	0 1 0 3 0 11	0.00 6.25 0.00 18.75 6.25 68.75

**Table 2:** Designs of studies included in the review (*n* = 15)

No.	First author (year)	Country	Study design	Age/mean/range of children	Sample size Intervention (I), Control (C)	Duration of intervention
1	Miller et al., 2025 [21]	USA	Secondary analysis of randomised trial	0–24 months	I = 201 C = 202	3 years
2	Gebretsadik et al., 2025 [22]	Ethiopia	Cluster randomised trial	6–23 months	I = 219 C = 219	12 months
3	Sosanya et al., 2025 [23]	Nigeria	Individually randomised controlled trial	0–2 years	I = 108 C = 108	6 months
4	Nix et al., 2024 [24]	USA	Individually randomised controlled trial	2 years	I = 123 C = 119	12 weeks
5	Ling et al., 2024 [25]	USA	Quasi-experimental pilot study	3–5 years	I = 107	14 weeks
6	Lee et al., 2023 [26]	USA	Randomised pilot study	1–3 years	I = 37 C = 36	8 weeks
7	Beck et al., 2023 [27]	USA	Mixed method randomised trial	0–1 month	I = 48 C = 48 I = 17 (semi-structured interview)	15 months
8	Wen et al., 2023 [28]	Australia	Pragmatic trial	2–3 years	I = 331 C = 331	24 months
9	Tovar et al., 2023 [29]	USA	Randomised pilot study	2–5 years	I = 33 C = 30	6 months
10	Hughes et al., 2021 [30] and Hughes et al., 2020 [31]	USA	Individually randomised controlled trial	3–5 years	I = 136 C = 119	7 weeks + 12 months follow-up
11	Nix et al., 2021 [32]	USA	Individually randomised controlled trial	18–36 months	I = 38 C = 35	10 weeks
12	Messito et al., 2020 [33]	USA	Individually randomised controlled trial	0–10 months	I = 202 C = 210	9 months
13	Fisher et al., 2019 [34]	USA	Individually randomised controlled trial	3–5 years	I = 59 C = 60	12 weeks
14	Fiks et al., 2017 [35]	USA	Individually randomised controlled trial	0–9 months	I = 43 C = 44	11 months
15	Sun et al., 2017 [36]	USA	Individually randomised controlled trial	3–5 years	I = 16 C = 16	8 weeks

The studies targeted diverse age ranges, generally spanning from 0 to 5 years, with the first 24 months representing the most common focus. The diversity of age ranges reflected recognition that intervention opportunities exist across the entire childhood period, consistent with evidence regarding the critical importance of this developmental window. The duration of interventions varied from seven weeks to 3 years for core delivery components, with some including extended follow-up periods. Most studies required participants be caregivers (typically mothers) of eligible young children rather than requiring child participation in intervention activities. Table 3 (see Additional File 1) summarises the intervention characteristics of the included studies according to the TIDieR checklist.

### Types of intervention domains and implementation strategies

Based on the synthesis of intervention domains and implementation strategies (Table 4 - see Additional File 2), the fifteen interventions were categorised into four domains: (A) parenting-focused, (B) home-based nutrition education and skill-building, (C) digital, and (D) multicomponent. These domains reflect the primary mechanisms through which positive parental feeding practices were targeted, rather than mutually exclusive delivery modes.

#### Parenting-focused

Seven interventions (46.67%) [21, 24, 25, 30–34] were under the parenting-focused domain, emphasising parental feeding styles, responsive feeding, autonomy support, scaffolding, and the quality of parent–child interactions during feeding. These interventions were delivered primarily through home visits [24, 32], clinic-based programmes [21], or group-based sessions [25, 30, 31, 33, 34] and consistently incorporated active skill-building through observation, coaching, modelling, and guided practice. Across this domain, interventions targeted multiple feeding constructs simultaneously, most commonly responsive feeding, feeding styles, feeding environment, autonomy support, and meal structure. Implementation strategies included video feedback of parent–child interactions [21, 30, 31, 33], real-time coaching during food preparation or meals [24, 32–34], experiential learning activities [21, 25, 30, 31], stress management [25] and coping strategies related to feeding, and structured curricula [24, 27] delivered by trained professionals or home visitors.

Outcome findings across the parenting-focused domain showed consistent improvements in parental feeding practices. Specifically, studies reported increases in responsive feeding practices, greater use of authoritative or autonomy-supportive feeding practices, and reductions in pressuring, indulgent, or controlling feeding practices. Several interventions also demonstrated improvements in child eating behaviours, such as enhanced self-regulation during meals, increased healthy eating habits, and improved fruit and vegetable intake. While some studies reported favourable changes in child dietary intake (e.g., reduced solid fat and added sugar consumption), short-term changes in child BMI were generally not observed.

#### Home-based nutrition education and skill-building

One intervention (6.67%) [22] was under the home-based nutrition education and skill-building domain, with a primary focus on complementary feeding practices, practical feeding skills, and household food routines [22]. This intervention relied on community-based delivery, with trained community health workers conducting repeated household visits and group discussions. The intervention emphasised demonstration-based learning, including preparation of complementary foods, guidance on meal frequency, portion size, and food consistency, and reinforcement of responsive feeding principles through repeated follow-up. Outcomes from this domain indicated improvements in dietary diversity, meal frequency, and maternal feeding knowledge, alongside increased alignment with age-appropriate complementary feeding recommendations.

#### Digital

Four interventions (26.67%) [23, 28, 35, 36] were under the digital domain, utilising mobile applications [23, 28], telephone-based coaching [28], SMS messaging [28], social media platforms [35], or tablet-based educational modules [36]. These interventions primarily targeted responsive feeding beliefs, feeding styles, and the feeding environment, with some also incorporating autonomy-supportive messaging. The interventions were typically implemented through asynchronous or remote delivery, including interactive app-based learning, quizzes with automated feedback, clinician- or nurse-led telephone coaching, moderated peer discussion groups, and multimedia educational content. These implementation strategies aimed to enhance accessibility and scalability, particularly for families facing structural barriers to in-person participation. Across studies, the digital domain consistently reported improvements in parental feeding knowledge, feeding beliefs, and self-efficacy, as well as reductions in specific non-responsive practices such as feeding to soothe or pressure during meals. Several studies also reported improvements in aspects of the feeding environment, including reduced screen use during meals and improved home food stimulus environments. However, effects on child anthropometric outcomes were minimal, and behavioural changes were more commonly cognitive or attitudinal in nature.

#### Multicomponent

Three interventions (20%) [26, 27, 29] were under the multicomponent domain, integrating parenting-focused with nutrition education, skill-building, and digital or system-level reinforcement. These interventions combined multiple delivery channels, including home visits [29], clinic-based counselling [27], digital platforms [26], text messaging [26, 27, 29], printed materials [29], and food provision [26] to support sustained behaviour change. The multicomponent domain consistently targeted responsive feeding, alongside meal structure, autonomy support, feeding styles, and the feeding environment. Implementation strategies emphasised repeated exposure to feeding messages, goal setting, personalised feedback, and opportunities for parents to practice feeding skills within their home environments. Outcome findings demonstrated improvements, including increases in responsive feeding practices, reductions in controlling feeding behaviours, improvements in home food environments, and enhanced meal structure. Several studies also reported improvements in child dietary behaviours, such as increased fruit intake or improved diet quality; however, changes in weight-related outcomes remained modest.

### Intervention outcomes and general findings

Across the fifteen intervention programmes [21–36], outcomes were most frequently reported at the level of parental feeding practices, followed by child eating and dietary behaviours, with anthropometric outcomes assessed less consistently. Overall, interventions demonstrated greater and more consistent effects on feeding-related behaviours and environments than distal weight-related outcomes.

### Parental feeding outcomes

Changes in parental feeding practices were the most commonly reported outcomes across domains. Improvements in responsive feeding, including greater recognition of child hunger and satiety cues and more appropriate parental responses were observed across parenting-focused, digital, and multicomponent domains [21, 26, 30, 31, 33, 35]. Several studies also reported reductions in non-responsive feeding practices, such as pressuring, restrictive, indulgent feeding, or feeding to soothe [21, 32, 34, 36]. Interventions that explicitly targeted feeding styles and autonomy support frequently reported shifts toward more authoritative or autonomy-supportive feeding practices, including increased use of guided choices, non-coercive language, and encouragement of child self-feeding [24, 27, 30, 31]. Increases in parental feeding knowledge and self-efficacy were also commonly reported, particularly in interventions incorporating educational or reflective components [23, 25, 36].

### Feeding environment and meal structure

Improvements in the feeding environment and meal structure were reported across multiple domains. These included more regular meal routines, increased shared family meals, reductions in screen use during eating occasions, and improvements in the availability and presentation of healthy foods within the home [25, 28, 29]. Interventions addressing parental stress or coping alongside feeding guidance reported additional benefits in perceived household food environments and parental capacity to maintain routines under stress [25].

### Child eating and dietary outcomes

Several interventions reported favourable changes in child eating behaviours and dietary intake, although these outcomes were less consistently assessed than parental feeding practices. Reported improvements included increased fruit and vegetable intake, greater dietary diversity, improved diet quality, and enhanced child self-regulation during eating [22, 24, 25, 27, 29, 34]. These outcomes were more frequently observed in parenting-focused and multicomponent domains that incorporated active skill-building and repeated opportunities for parents to practice feeding strategies in everyday contexts.

### Anthropometric outcomes

In contrast to feeding-related outcomes, anthropometric measures such as child BMI or weight status were assessed in relatively few interventions and generally showed no significant short-term changes [30, 34, 36]. Where reported, null findings in weight outcomes occurred despite concurrent improvements in parental feeding practices or child dietary behaviours. Overall, the findings indicate that interventions targeting positive parental feeding practices in low-income households were the most effective at influencing parental behaviours, feeding-related beliefs, and the feeding environment, with more variable effects on child dietary behaviours and a limited short-term impact on anthropometric outcomes. Responsive feeding emerged as the most consistently improved construct across domains, while changes in feeding styles, autonomy support, and meal structure appeared to depend on intervention intensity and the inclusion of active skill-building components.

### Household Food Insecurity (HFI) in intervention design

Across the fifteen interventions included in this scoping review, explicit consideration of household food insecurity (HFI) within intervention design was limited, as shown in Table 3. Only three [25, 33, 35] interventions explicitly incorporated HFI-related elements, and two [26, 29] interventions implicitly addressed it, while the majority treated low-income status as a background characteristic without direct reference to food insecurity. Two interventions that were also included in the scoping review by Baxter et al. [15], Fiks et al. [35] and Messito et al. [33] both explicitly acknowledged household food insecurity. In the Grow2Gether trial, Fiks et al. [35] conceptualised food insecurity as a contextual factor influencing parental feeding behaviours, particularly the use of food to soothe infants. Similarly, the Starting Early Program evaluated by Messito et al. [33] measured food insecurity and situated responsive feeding guidance within the realities of constrained food access among low-income families.

**Table 3:** Consideration of HFI in intervention studies (*n* = 15)

No.	Author (year)	Explicit consideration of HFI	Implicit consideration of HFI	How HFI was addressed or positioned
1	Miller et al., 2025 [21]	No	No	NA
2	Nix et al., 2024 [24]	No	No	NA
3	Ling et al., 2024 [25]	Yes	-	HFI measured as an outcome; intervention addressed parental stress, coping, and home routines that may influence food security
4	Nix et al., 2021 [32]	No	No	NA
5	Hughes et al., 2021 [30] and Hughes et al., 2020 [31]	No	No	NA
6	Messito et al., 2020 [33]	Yes	-	Food insecurity measured and discussed as a contextual factor influencing feeding practices
7	Fisher et al., 2019 [34]	No	No	NA
8	Gebresadik et al., 2025 [22]	No	No	NA
9	Sosanya et al., 2025 [23]	No	No	NA
10	Wen et al., 2023 [28]	No	No	NA
11	Fiks et al., 2017 [35]	Yes	-	Conceptualised food insecurity as influencing feeding to soothe; explicitly discussed in intervention rationale
12	Sun et al., 2017 [36]	No	No	NA
13	Lee et al., 2023 [26]	-	Yes	Provided fruits, vegetables, and cooking utensils, indirectly addressing food access constraints
14	Beck et al., 2023 [27]	No	No	NA
15	Tovar et al., 2023 [29]	-	Yes	Targeted food availability, meal routines, and diet quality; acknowledged structural barriers without measuring HFI

Among the additional interventions identified in the present review, the study conducted by Ling et al. [25] was the only investigation that explicitly measured household food insecurity as an outcome. In this parenting-focused intervention, improvements in feeding practices were accompanied by reductions in reported food insecurity, suggesting potential spillover benefits of addressing parental stress, coping strategies, and home meal routines. Two further interventions addressed food insecurity implicitly rather than explicitly. Lee et al. [26] incorporated food provision through the distribution of fresh fruits, vegetables, and cooking utensils alongside digital feeding education, indirectly addressing food access constraints. Similarly, Tovar et al. [29] targeted home food availability, structured meal routines, and diet quality through home visits and tailored materials, acknowledging structural barriers to healthy eating without directly measuring food insecurity.

In contrast, the remaining interventions, including those employing parenting-focused, digital, and multicomponent domains, did not explicitly reference or measure household food insecurity. These interventions primarily focused on modifying parental feeding behaviours, interaction quality, and feeding environments, assuming socioeconomic disadvantage as a contextual backdrop rather than a central intervention target. Overall, while food insecurity was central to the conceptual framing of earlier interventions identified by Baxter et al. [15], most contemporary interventions included in this review did not explicitly integrate HFI into their design or evaluation. Instead, newer studies tended to prioritise the behavioural and relational components of feeding within low-income contexts, with food insecurity addressed indirectly or not at all.

## Discussion

This scoping review aimed to determine the key concepts of existing intervention programmes that employed positive parental feeding practices in low-income households, with a primary focus on the types of intervention and implementation strategies used. The findings provide a structured overview of how existing interventions conceptualise and operationalise feeding-related components, particularly responsive feeding, feeding styles, autonomy support, meal structure, and the feeding environment. Overall, the review highlights substantial conceptual convergence across interventions, alongside meaningful variations in implementation strategies, intensity, and outcome patterns.

### Centrality of responsive feeding across interventions

Responsive feeding was the most consistently targeted construct across all intervention types. Parenting-focused, digital, home-based, and multicomponent domains all sought to enhance caregivers’ ability to recognise and respond appropriately to children’s hunger and satiety cues while discouraging pressuring, restrictive, or emotionally driven feeding practices [21, 30, 31, 33, 35]. These findings align with theoretical models that position responsive feeding as a core mechanism linking parental behaviour to children’s self-regulation and eating behaviours during early childhood [37]. Importantly, improvements in responsive feeding were observed even in interventions that did not explicitly label their implementation strategy as “responsive feeding,” suggesting that the construct is frequently embedded within broader parenting, nutrition education, or obesity prevention frameworks [25, 29, 32]. This convergence supports the relevance of responsive feeding across diverse cultural, socioeconomic, and implementation contexts, including settings characterised by material and resource constraints.

### Intervention components and mechanisms of change

The parenting-focused domain was the most prevalent intervention and demonstrated the most consistent improvements in parental feeding practices. This domain typically incorporated active skill-building strategies, such as real-time coaching, video feedback, modelling of feeding interactions, and guided practice during food preparation or meals [21, 24, 34]. Such implementation strategies were associated with reductions in pressuring and controlling feeding practices and increases in autonomy-supportive and authoritative feeding practices [30, 31, 33]. The consistency of these findings suggests that interventions that directly target parent–child interaction quality may be particularly effective in shifting established feeding practices, even in the context of socioeconomic disadvantage.

In contrast, the digital domain primarily influenced parental feeding knowledge, beliefs, and self-efficacy [23, 35, 36]. While some reported reductions in non-responsive practices, such as feeding to soothe [35], changes in observed feeding practices and child outcomes were more variable. These findings indicate that, although the digital domain enhances accessibility and scalability, information-based or reflective learning alone may be insufficient to produce sustained behavioural change without opportunities for practice, coaching, or personalised feedback.

The multicomponent domain, which integrated parenting-focused with nutrition education, environmental modification, and digital reinforcement, demonstrated the broadest range of positive outcomes [26, 27, 29]. These programmes commonly reported improvements in responsive feeding, meal structure, and the home food environment, as well as favourable changes in child dietary behaviours. In particular, some of the interventions under this domain indirectly addressed structural constraints through food provision or improvements in food availability. These findings indicate practical pathways for adapting feeding interventions to contexts of economic hardship. However, such interventions were also more resource-intensive, raising considerations about feasibility, scalability, and sustainability in low-income settings.

### Feeding environment, meal structure, and household food insecurity

Although responsive feeding dominated intervention construct, meal structure and the broader feeding environment were less consistently operationalised as primary mechanisms of change. When explicitly addressed, interventions reported improvements in regular meal routines, reduced screen use during meals, and enhanced availability of healthy foods at home [25, 28, 29]. Given the structural constraints faced by low-income households, including the heightened risk of household food insecurity, these findings suggest that environmental and routine-based strategies may serve as underutilised yet critical levers for supporting responsive feeding in everyday contexts. Consistent with observations by Baxter et al. [15], relatively few interventions explicitly integrated household food insecurity into their theoretical framing or evaluation. In the present review, only a small number of studies measured food insecurity directly or addressed it implicitly through food provision, stress reduction, or improvements in food availability. Most interventions treated low-income status as a contextual backdrop rather than explicitly examining how food insecurity shapes parental feeding decisions. Recent interventions prioritised behavioural and relational components, often without directly addressing food insecurity.

### Autonomy support as an emerging but inconsistently defined construct

Autonomy support, which includes guided choice, encouragement of self-feeding, and child-led intake, was inconsistently defined and measured across studies, despite being central to several interventions [24, 27, 34]. While autonomy-supportive practices are theoretically aligned with responsive feeding and self-regulation, greater conceptual clarity is needed regarding how autonomy support can be operationalised within the constraints of low-income and food-insecure contexts, where food choice and availability may be limited.

### Limited effects on anthropometric outcomes

Consistent with prior early childhood intervention research, this review found limited short-term effects on child weight or BMI, even among interventions that demonstrated improvements in parental feeding practices and child dietary behaviours [34, 37]. This pattern likely reflects the multifactorial determinants of child growth, the young age of participants, and relatively short follow-up periods. It may also reflect the limited extent to which interventions addressed broader structural determinants of diet and growth, such as food insecurity. These findings highlight the importance of viewing feeding practices, meal routines, and the home food environment as meaningful intermediate outcomes, rather than relying solely on anthropometric change as an indicator of intervention success. This is also supported by broader systematic reviews, suggesting weight status may not be a sensitive short-term outcome for behavioural-focused feeding interventions [37, 38] and that associations between parental feeding practices and child weight status are complex and often bidirectional, with coercive practices such as restriction and pressure to eat showing inconsistent relationships with BMI and obesity risk [39].

#### Implications for intervention design and delivery

**Fig. 2:**
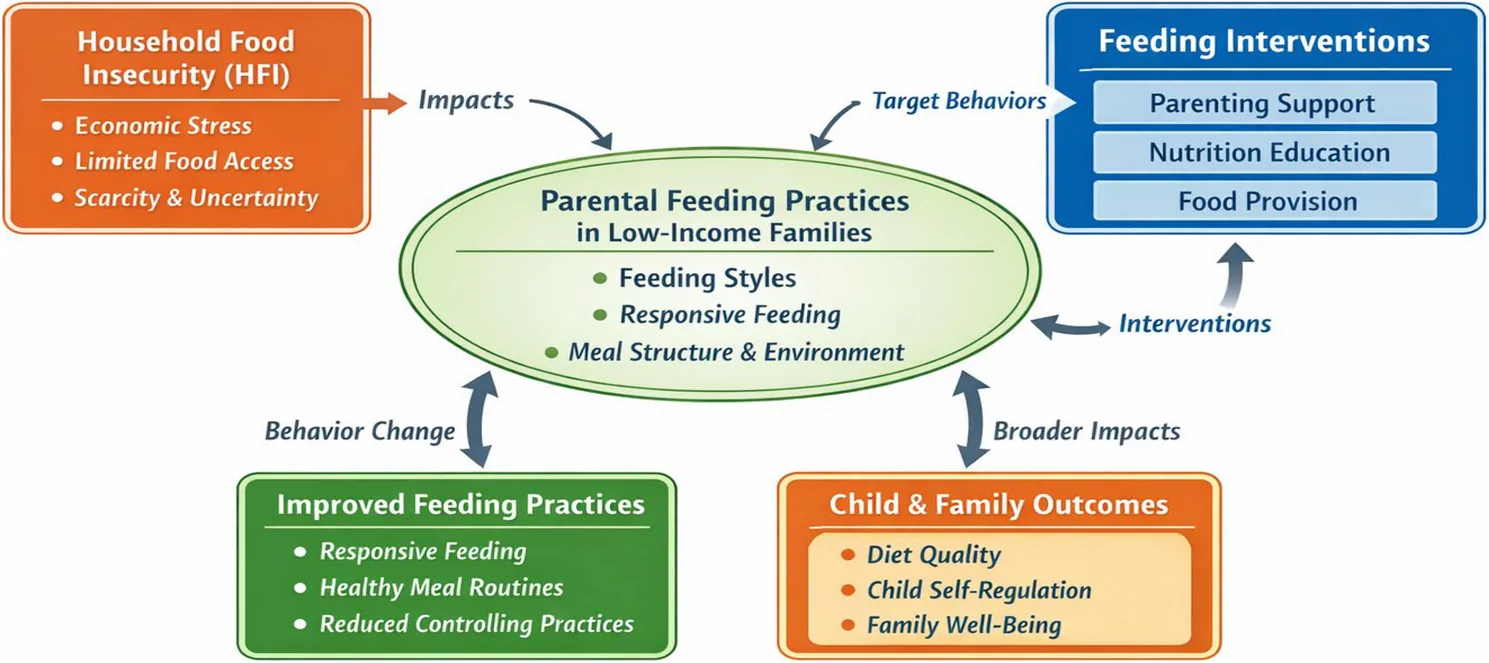
Conceptual framework of future feeding interventions

Guided by the conceptual framework developed in this review, as illustrated in Fig. 2, several recommendations for the design of future feeding interventions in low-income households can be identified. First, interventions should continue to prioritise responsive feeding as a central mechanism of change, given its consistent presence across intervention types and its robust association with improvements in parental feeding practices and child eating behaviours. However, responsive feeding guidance should move beyond didactic messaging to incorporate active skill-building strategies, such as real-time coaching, modelling, and guided practice, which are more consistently associated with behavioural change than information-based implementation strategies alone.

Second, the framework highlights the importance of embedding feeding interventions within a broader contextual understanding of household food insecurity (HFI). Although few interventions explicitly addressed HFI, those that incorporated food provision, stress management, or improvements in home food availability demonstrated potential pathways for aligning feeding guidance with families’ lived constraints. Future interventions should therefore consider integrating responsive feeding strategies with structural supports, such as food access resources, budgeting assistance, or linkage to food assistance programmes, to enhance feasibility and sustainability in food-insecure contexts [40].

Third, interventions should more deliberately target meal structure and the feeding environment as complementary mechanisms supporting responsive feeding [41]. Regular meal routines, reduced screen use during meals, and improved home food availability emerged as underutilised yet influential components, particularly in low-income settings where environmental cues strongly shape feeding interactions. Designing interventions that explicitly address these contextual features may support caregivers’ capacity to implement responsive feeding practices consistently, even under conditions of stress or resource limitation.

Fourth, autonomy-supportive feeding practices warrant greater conceptual clarity and intentional integration into intervention design. While autonomy support was frequently referenced [42], its operationalisation varied widely, and few interventions explicitly examined how autonomy can be supported when food choice is constrained. Future programmes should articulate developmentally appropriate, context-sensitive, and autonomy-supportive strategies, such as guided choice within available foods and encouragement of self-regulation, which are feasible for households experiencing economic hardship.

Finally, the conceptual framework reinforces the importance of selecting appropriate outcome indicators for evaluating intervention success. Given the limited and inconsistent effects observed across studies on anthropometric outcomes, future interventions should prioritise feeding practices, meal routines, and the home food environment as meaningful intermediate outcomes [43, 44]. These proximal indicators may be more sensitive to change, more closely aligned with intervention components, and more relevant to families’ day-to-day feeding experiences, particularly in the context of household food insecurity. Together, these recommendations suggest that feeding interventions for low-income families may be most effective when they are relational, skill-based, and context-responsive, explicitly acknowledging the structural conditions under which feeding occurs while supporting caregivers’ capacity to engage in positive feeding practices.

### Strengths and limitations

This review provides a comprehensive synthesis of intervention components and outcomes related to positive parental feeding practices in low-income households. However, heterogeneity in intervention design, outcome measures, and follow-up duration limited direct comparison across studies. Additionally, many studies relied on parent-reported feeding measures, which may be subject to social desirability or recall bias. Importantly, while all included studies targeted populations at elevated risk of household food insecurity, relatively few interventions explicitly measured or incorporated food insecurity into their intervention design or evaluation. As a result, the extent to which intervention components were responsive to structural food access constraints could not be fully assessed. As a scoping review, this study did not evaluate intervention effectiveness or risk of bias; findings should therefore be interpreted as descriptive and exploratory rather than evaluative.

Future interventions should prioritise greater conceptual clarity and measurement consistency in the operationalisation of positive parental feeding practices, particularly for autonomy support, meal structure, and the feeding environment. Explicit articulation of theory-driven pathways linking intervention components to child outcomes would strengthen intervention design and evaluation. Given the limited impact on anthropometric outcomes, future research should consider longer follow-up periods and prioritise feeding practices as primary intermediate outcomes. Integrating feeding-focused interventions with strategies that address structural stressors in low-income households, such as food insecurity, caregiver stress, and time constraints, may enhance intervention relevance, equity, and long-term effectiveness. Finally, future interventions should more explicitly integrate household food insecurity into their theoretical frameworks, either by measuring food insecurity as a moderator of intervention effects or by embedding feeding guidance within broader food access and economic support strategies. Aligning responsive feeding interventions with structural supports may enhance their relevance and sustainability in low-income contexts.

## Conclusions

This scoping review synthesised evidence from 16 studies representing 15 unique intervention programmes that employed positive parental feeding practices among low-income households. The findings demonstrate substantial conceptual alignment across interventions, with responsive feeding emerging as the central and most consistently targeted construct, regardless of implementation mode or setting. Parenting-focused and multicomponent domains that incorporated active skill-building, repeated contact, and contextual support showed the most consistent improvements in parental feeding practices and aspects of the feeding environment. In contrast, the digital domain primarily influenced parental knowledge, beliefs, and self-efficacy, with more variable effects on observed feeding practices. Across interventions, changes in parental feeding practices were more robust and consistent than changes in child anthropometric outcomes, highlighting the importance of viewing feeding practices and environments as meaningful intermediate outcomes in early childhood. Future feeding interventions should prioritise skill-based, context-responsive strategies that align behavioural guidance with the realities of food insecurity. Strengthening conceptual clarity, implementation intensity, and integration with broader structural supports may enhance the relevance and sustainability of feeding interventions in low-income populations.

## Supplementary Information

Additional file 1.

Additional file 2.

Additional file 3.

## Data Availability

All data relevant to the results of this review are available in the main tables. The data collected, including data extraction forms, can be made available upon reasonable request.

## Abbreviations

HFI: Household Food Insecurity
BMI: Body Mass Index
TIDieR: Template for Intervention Description and Replication
