# Supplementary material for: Interventions for positive parental feeding practices in low-income households: a scoping review
Source: Int J Behav Nutr Phys Act. 2026 Jun 17;23:83. doi: 10.1186/s12966-026-01938-5 (PMC13543349; doi:10.1186/s12966-026-01938-5)
Supplement: Supplementary file 1 [file 12966_2026_1938_MOESM1_ESM.docx]

**Table 3** Intervention characteristics of included studies (n=15) according to the TIDieR	checklist

| **Study** | **TIDieR Element** | |
| --- | --- | --- |
| Miller et al., 2025 [21] | Brief name: | The Smart Beginnings intervention (SB) on feeding styles via earlier intervention effects on cognitive stimulation |
|  | Why (rationale, theory or goal): | This intervention aimed to enhance early childhood development and school readiness through improved parenting practices, particularly cognitive stimulation and responsive parenting |
|  | What (programme content or type): | Tiered intervention integrating PlayReadVIP (primary care-based parenting program) and Family Check-Up (FCU) (home-based parenting support). Activities included book/ toy provision, video-recorded of parent-child interaction and parenting training and motivational interviewing |
|  | Who (programme provider): | Interventionists |
|  | How (delivery mode and setting): | Mixed delivery during pediatric primary care clinic sessions and home visits |
|  | Where (delivery platform): | Pediatric primary care and participants’ homes |
|  | When and how much: | i) Programme commencement: from birth to 24 months  ii) How often-programme frequency: 14 sessions  iii) Length of session: 25-30 minutes  iv) Programme duration: 3 years |
|  | Tailoring: | Intervention was adaptive and tailored based on child behaviour, family wellbeing, and caregiving risk |
|  | Modifications: | ?*  * Information about the element was not reported/ not sufficiently reported |
|  | How well: | ?*  * Information about the element was not reported/ not sufficiently reported |
| Gebretsadik et al., 2025 [22] | Brief name: | Behaviour Change Communication (BCC) intervention via Women Development Army |
|  | Why (rationale, theory or goal): | The intervention aimed to improve complementary feeding practices and maternal knowledge in low-resource settings |
|  | What (programme content or type): | Five-session BCC package including nutrition education, complementary feeding practices, hygiene practices, food preparation techniques and practical demonstrations based on WHO/UNICEF materials |
|  | Who (programme provider): | Trained community volunteers (Women Development Army) |
|  | How (delivery mode and setting): | Face-to-face delivery through home visits, demonstrations, group discussions and visual aids (posters and flip charts) |
|  | Where (delivery platform): | Delivered at community/ home-based settings in rural villages |
|  | When and how much: | i) Programme commencement: from 6 – 23 months  ii) How often-programme frequency: biweekly (first 3 months), monthly (next 3 months) and every 2 months (last 6 months)  iii) Length of session: 5 modules, each lasting 3 days  iv) Programme duration: 12 months |
|  | Tailoring: | Limited or implicit. Some adaptation made through use of locally available foods and community-based delivery. |
|  | Modifications: | ?*  * Information about the element was not reported/ not sufficiently reported |
|  | How well: | ?*  * Information about the element was not reported/ not sufficiently reported |
| Sosanya et al., 2025 [23] | Brief name: | BabyThrive, a mobile-based application intervention |
|  | Why (rationale, theory or goal): | This intervention aimed to improve infant and young child feeding (IYCF) knowledge among teenage mothers using scalable mHealth solutions |
|  | What (programme content or type): | Mobile-based role play video game including breastfeeding education, complementary feeding guidance, 30 nutritionally adequate recipes using local foods and interactive learning modules |
|  | Who (programme provider): | Research assistants |
|  | How (delivery mode and setting): | Mobile app (self-directed learning) supported by initial training, follow-up phone calls and monitoring visits |
|  | Where (delivery platform): | Delivered using smartphone-based (offline functionality) |
|  | When and how much: | Information available from: Sosanya ME, Samuel FO, Bashir S, Omoera VO, Freeland-Graves JH. A Mobile Gaming App to Train Teenage Mothers on Appropriate Child Feeding Practices: Development and Validation Study. *J Med Internet Res*. 2024;26:e53560. doi:10.2196/53560  i) Programme commencement: from birth to 2 years  ii) How often-programme frequency: ?*  iii) Length of session: ?*  iv) Programme duration: 6 months  * Information about the element was not reported/ not sufficiently reported |
|  | Tailoring: | Limited tailoring, app available in local language |
|  | Modifications: | ?*  * Information about the element was not reported/ not sufficiently reported |
|  | How well: | ?*  * Information about the element was not reported/ not sufficiently reported |
| Nix et al., 2024 [24] | Brief name: | Recipe 4 Success intervention embedded within Early Head Start (Scaled-up study) |
|  | Why (rationale, theory or goal): | This intervention aimed to promote toddler self-regulation, responsive food parenting and healthy eating habits |
|  | What (programme content or type): | Structured cooking-based parenting intervention including weekly guided food preparation lessons, parenting skill coaching, mindfulness and gratitude before eating, physical activity for toddlers and weekly reflection and planning |
|  | Who (programme provider): | Home visitors (trained intervention staff) |
|  | How (delivery mode and setting): | Face-to-face home visits, coaching-based, interactive sessions during real-life cooking activities |
|  | Where (delivery platform): | Delivered at participants’ homes |
|  | When and how much: | i) Programme commencement: toddler aged 2 years  ii) How often-programme frequency: 10 core cooking sessions and weekly home visits  iii) Length of session: 45 minutes  iv) Programme duration: 12 weeks |
|  | Tailoring: | Tailored, adapted based on food allergies and cooking facilities. Recipes and activities adjusted to family context |
|  | Modifications: | ?*  * Information about the element was not reported/ not sufficiently reported |
|  | How well: | Intervention fidelity was assessed via digital audio recordings of entire home visits. The recordings were collected by the home visitors, but rated by a team of a laboratory-based research assistants. Extensive ratings of the quality of delivery demonstrated that almost all lessons were high in educational content, home visitor therapeutic skills, home visitor-parent working alliance, and parent and toddler engagement |
| Ling et al., 2024 [25] | Brief name: | Happy Family, Healthy Kids, a healthy eating and stress management intervention |
|  | Why (rationale, theory or goal): | This intervention aimed to improve diet quality, stress coping and mental health, and reduce emotional eating and obesity risk based on the actor-partner interdependence model, social cognitive theory, allostatic load model and transactional theory of stress and coping |
|  | What (programme content or type): | Multicomponent programme including weekly educational videos, fylers with behaviour change strategies, weekly habit formation tasks, parent-child activities, yoga sessions and motivational text messages |
|  | Who (programme provider): | Trained Head Start teachers or interventionists |
|  | How (delivery mode and setting): | Hybrid digital delivery through social media/ website-based learning, zoom meetings, and text messaging |
|  | Where (delivery platform): | Delivered via online platforms such as Facebook (private website), zoom and messaging platforms |
|  | When and how much: | i) Programme commencement: child of aged 3-5 years  ii) How often-programme frequency: 3 motivational text messages per week, 3 virtual meetings (Weeks 1, 7 and 14) and weekly videos for 14 weeks  iii) Length of session: 30 minutes  iv) Programme duration: 14 weeks |
|  | Tailoring: | Limited, some personalization through parent engagement, interactive tasks with no explicit individualised adaptation protocol |
|  | Modifications: | ?*  * Information about the element was not reported/ not sufficiently reported |
|  | How well: | From 84 parents, 86.9% used the programme cookbook at least once a week, about 88.1% agreed that the programme has helped them to practice healthy lifestyle and 89.3% would use the materials in the future. |
| Lee et al., 2023 [26] | Brief name: | Theory-based, multicomponent eHealth obesity prevention intervention |
|  | Why (rationale, theory or goal): | This intervention aimed at improving child health behaviours and parental psychosocial attributes and feeding practices, underpinned by Social Cognitive Theory |
|  | What (programme content or type): | Multicomponent intervention including educational videos, cooking tutorials, goal setting, self-monitoring and reflection, text message reminders and provision of fresh fruits and vegetables |
|  | Who (programme provider): | Research team |
|  | How (delivery mode and setting): | Delivered through website-based learning, mobile/ text-based support, provision of fresh fruits and vegetables biweekly and self-directed with guidance |
|  | Where (delivery platform): | Delivered via online platform and home-based participation |
|  | When and how much: | i) Programme commencement: children aged between 1-3 years  ii) How often-programme frequency: weekly (educational videos and text reminders), and biweekly food provision of fresh fruits and vegetables  iii) Length of session: ?*  iv) Programme duration: 8 weeks  * Information about the element was not reported/ not sufficiently reported |
|  | Tailoring: | Tailored based on personalised goal setting, self-reflection and feedback based on participant responses |
|  | Modifications: | ?*  * Information about the element was not reported/ not sufficiently reported |
|  | How well: | ?*  * Information about the element was not reported/ not sufficiently reported |
| Beck et al., 2023 [27] | Brief name: | *Futuros Fuertes* (Strong Futures) Intervention |
|  | Why (rationale, theory or goal): | This intervention aimed to promote healthy feeding, screen time, and sleep practices among low-income Latino infants based on the Information-Motivation-Behavioural Skills (IMB) model of health behaviour change |
|  | What (programme content or type): | The intervention was delivered through individual health education sessions from a bilingual, bicultural lay health educator in the primary care clinic and two text messages per week |
|  | Who (programme provider): | Lay health educator trained by researcher |
|  | How (delivery mode and setting): | Face-to-face sessions after child visit, text messaging and telephone delivery |
|  | Where (delivery platform): | Delivered at primary care clinic, via text messages and via telephone |
|  | When and how much: | i) Programme commencement: infant aged less than one month  ii) How often-programme frequency: 2 text messages per week from infant age 2 weeks to 15 months, individual health education sessions at infant aged 2 weeks, at 2, 4, 6, 9 and 12 months  iii) Length of session: 20-25 minutes of individual health education sessions  iv) Programme duration: 15 months |
|  | Tailoring: | Culturally tailored for Spanish-speaking Latino parents, iterative refinement using parent feedback and developmentally appropriate messaging |
|  | Modifications: | Shifted from face-to-face session to telephone delivery due to COVID-19 |
|  | How well: | Sessions recorded and reviewed by principal investigator and ongoing feedback to educators. Dose measured on number of completed sessions and texts received |
| Wen et al., 2023 [28] | Brief name: | Nurse-led telephone-based early childhood obesity prevention intervention |
|  | Why (rationale, theory or goal): | Based on the Health Belief Model, this intervention aimed to reduce obesity risk via behaviour change in diet, activity, and screen time |
|  | What (programme content or type): | Intervention period started with a mailout of an intervention booklet, followed by a telephone support call and SMS. |
|  | Who (programme provider): | Child and family health nurses |
|  | How (delivery mode and setting): | Delivered through telephone-based support, two-way SMS system and telehealth (use of telecommunication technologies) |
|  | Where (delivery platform): | Delivered through home-based via telephone and SMS |
|  | When and how much: | i) Programme commencement: children aged 2 - 3 years  ii) How often-programme frequency: 5 telephone-based sessions, twice a week of SMS  iii) Length of session: 5 telephone-based sessions (duration 45-60 minutes), SMS (twice a week for 4 weeks at 10am- 1pm),  iv) Programme duration: 24 months |
|  | Tailoring: | Some flexibility during calls (discussion-based support) |
|  | Modifications: | The original study protocol can be found from Wen LM, Rissel C, Xu H, et al. Linking two randomised controlled trials for Healthy Beginnings©: optimising early obesity prevention programs for children under 3 years. *BMC Public Health*. 2019;19:739. https:// doi.org/10.1186/s12889-019-7058-9.  Calls extended in duration due to COVID-19 as additional discussion needed on related issues such as measures of protection and immunisation. Also, this study was an extension of the original study protocol, in which two telephone-based intervention sessions at 38-40 and 42-44 months of child age in addition to three intervention sessions delivered in the original trial |
|  | How well: | Intervention engagement monitored on completion of calls and SMS messages sent and process evaluation conducted will be reported elsewhere |
| Tovar et al., 2023 [29] | Brief name: | Strong Families Start at Home Intervention |
|  | Why (rationale, theory or goal): | This intervention aimed to empower parents from low-income, predominantly Hispanic/ Latinx families to identify and implement responsive food parenting practices, tailor their practices to their child’s appetitive traits and utilise healthy food shopping and preparation strategies |
|  | What (programme content or type): | Six visits with a community health worker trained in motivational interviewing, in-home cooking and reading activities, personalised feedback on a recorded family meal, text messages and tailored printed materials |
|  | Who (programme provider): | Community health worker |
|  | How (delivery mode and setting): | Delivered through home-based (in-person and by phone) and video feedback approach |
|  | Where (delivery platform): | Delivered at home setting and virtual (FaceTime/ WhatsApp) |
|  | When and how much: | i) Programme commencement: child of aged between 2 and 5 years  ii) How often-programme frequency: 3 home visits, 3 phone calls, 2 text messages per week  iii) Length of session: home visits (60-75 minutes), phone calls (30-45 minutes)  iv) Programme duration: 6 months |
|  | Tailoring: | Tailored to child appetitive traits and personalised feedback from meal videos |
|  | Modifications: | Converted to virtual delivery during COVID-19 and cooking session replaced with food boxes and video tutorials |
|  | How well: | Intervention fidelity was measured using audio recordings, checklists for home visits, phone calls, goal setting, planning documentation, participant satisfaction surveys, completion of the video recording of a family meal and qualitative interviews across five domains recommended in the National Health Behaviour Change Consortium |
| Hughes et al., 2021 [30] and Hughes et al., 2020 [31] | Brief name: | The SEEDS (Strategies for Effective Eating Development) obesity prevention programme |
|  | Why (rationale, theory or goal): | Designed to prevent childhood obesity by improving parental feeding practices, child self-regulation, executive functioning, and willingness to try new foods, particularly among low-income Hispanic families |
|  | What (programme content or type): | Structured curriculum with seven core lessons including topics on hunger/ fullness cues, portion sizes, family routines, trying new foods and managing external influences. Besides that, the SEEDS programme also involved video-based learning and experiential group activities |
|  | Who (programme provider): | Trained facilitators |
|  | How (delivery mode and setting): | Conducted through group-based sessions and family-based participation |
|  | Where (delivery platform): | Delivered through group setting |
|  | When and how much: | Information available from Hughes SO, Power TG, Beck A, et al. Strategies for Effective Eating Development-SEEDS: Design of an Obesity Prevention Program to Promote Healthy Food Preferences and Eating Self-Regulation in Children From Low-Income Families. *J Nutr Educ Behav*. 2016;48(6):405-418.e1. doi:10.1016/j.jneb.2016.04.388  i) Programme commencement: from 3-5 years  ii) How often-programme frequency: 7 weekly sessions for parent, child and family  iii) Length of session: ?*  iv) Programme duration: 7 weeks + 12 months follow-up  * Information about the element was not reported/ not sufficiently reported |
|  | Tailoring: | Used of culturally relevant materials and adjusted for literacy levels during implementation |
|  | Modifications: | ?*  * Information about the element was not reported/ not sufficiently reported |
|  | How well: | High implementation fidelity through observer checklist. Across the parent, child, and family lessons, fidelity ranged from 67% to 100% with a mean of 95%. Despite this wide range, 85% of the lessons had fidelity values greater than 85% |
| Nix et al., 2021 [32] | Brief name: | Recipe 4 Success Intervention (Initial study) |
|  | Why (rationale, theory or goal): | Designed to improve toddlers’ healthy eating habits and self-regulation, and enhance responsive feeding and sensitive scaffolding, particularly among low-income families |
|  | What (programme content or type): | Ten structured weekly lessons on didactic education (feeding, sleep and development), hands-on food preparation activities, active coaching on parenting skills and skill generalisation to daily routines |
|  | Who (programme provider): | Early Head Start home visitors |
|  | How (delivery mode and setting): | Delivered during home visits and integrated into existing Early Head Start programme |
|  | Where (delivery platform): | Delivered through home setting |
|  | When and how much: | i) Programme commencement: from 18-36 months  ii) How often-programme frequency: 10 consecutive weekly sessions  iii) Length of session: 45 minutes  iv) Programme duration: 10 weeks |
|  | Tailoring: | Activities adapted to toddlers’ developmental skill levels and adjusted for food allergies, equipment and home context |
|  | Modifications: | ?*  * Information about the element was not reported/ not sufficiently reported |
|  | How well: | Home visitors rated fidelity after each session using 15-item scale |
| Messito et al., 2020 [33] | Brief name: | Starting Early Program (StEP) intervention |
|  | Why (rationale, theory or goal): | Based on application of Social Learning Theory, this intervention aimed to prevent obesity through promotion of feeding practices starting from pregnancy in low-income Hispanic families |
|  | What (programme content or type): | Delivery of individual nutrition counselling focused on prenatal and postpartum and Nutrition and Parenting Support Group (NPSG) that involved topics on breastfeeding, responsive feeding, portion sizes and parenting |
|  | Who (programme provider): | Trained health educators/ Registered Dietitians (RDs) |
|  | How (delivery mode and setting): | Combination of individual counselling and group sessions that delivered alongside routine primary care visits with interactive discussion and demonstration |
|  | Where (delivery platform): | Delivered at prenatal and pediatric primary care clinics |
|  | When and how much: | i) Programme commencement: third trimester of pregnancy  ii) How often-programme frequency: 2 individual sessions at prenatal and postpartum, and 5 group sessions at 1, 2, 4, 6 and 9 months of child age  iii) Length of session: ?*  iv) Programme duration: 9 months  * Information about the element was not reported/ not sufficiently reported |
|  | Tailoring: | ?*  * Information about the element was not reported/ not sufficiently reported |
|  | Modifications: | ?*  * Information about the element was not reported/ not sufficiently reported |
|  | How well: | Fidelity was assessed during 57% of the NPSG sessions. Of these sessions assessed, 94% provided all curriculum components |
| Fisher et al., 2019 [34] | Brief name: | Food, Fun, and Families (FFF) Intervention |
|  | Why (rationale, theory or goal): | Based on authoritative food parenting theory, this intervention aimed to reduce children’s intake of solid fats and added sugar (SoFAS) through structure and autonomy-supportive feeding practices |
|  | What (programme content or type): | Twelve structured weekly group sessions that involved parenting skills, behaviour change, environmental strategies and interactive demonstrations |
|  | Who (programme provider): | Graduate-level interventionists trained by clinical psychologists |
|  | How (delivery mode and setting): | Delivered through face-to-face group-based sessions |
|  | Where (delivery platform): | Delivered at university clinic-based setting |
|  | When and how much: | i) Programme commencement: between 3 and 5 years  ii) How often-programme frequency: 12 weekly sessions  iii) Length of session: 60 minutes  iv) Programme duration: 12 weeks |
|  | Tailoring: | Developed using formative research that aligned with mothers’ values (bonding and child behaviour) and allowed some individual adaptation |
|  | Modifications: | ?*  * Information about the element was not reported/ not sufficiently reported |
|  | How well: | ?*  * Information about the element was not reported/ not sufficiently reported |
| Fiks et al., 2017 [35] | Brief name: | Grow2Gether peer group Intervention |
|  | Why (rationale, theory or goal): | Underpinned by Social Learning Theory, this intervention aimed to improve infant feeding and obesity-related behaviours via peer support and social media engagement |
|  | What (programme content or type): | Private Facebook peer group that delivered video-based currriculum on topics such as feeding, sleep, parenting and maternal well-being |
|  | Who (programme provider): | Psychologist facilitator |
|  | How (delivery mode and setting): | Delivered through online peer-group (Facebook) |
|  | Where (delivery platform): | Delivered using digital platform (private Facebook group) |
|  | When and how much: | i) Programme commencement: 2 months of prenatal  ii) How often-programme frequency: weekly videos then biweekly  iii) Length of session: infant feeding practices (11 weeks), sleep (7 weeks), positive parenting (12 weeks), and maternal well-being (8 weeks)  iv) Programme duration: until infant aged 9 months (11 months in total from programme commencement) |
|  | Tailoring: | Content matched to infant developmental stages |
|  | Modifications: | ?*  * Information about the element was not reported/ not sufficiently reported |
|  | How well: | ?*  * Information about the element was not reported/ not sufficiently reported |
| Sun et al., 2017 [36] | Brief name: | Home-based and technology-centered childhood obesity prevention intervention |
|  | Why (rationale, theory or goal): | This intervention aimed to improve health behaviours of low-income, overweight, or obese Chinese mothers and their children based on the Information-Motivation-Behaviour (IMB) model |
|  | What (programme content or type): | The intervention consisted of eight weekly educational modules accessed online via tablet computers that covered topics such as introduction to the 5-4-3-2-1-0 programme, energy balance, what to feed my family, grocery shopping, find fun in physical activity, less sit, more fit, smart parenting and maintain a healthy weight for life. The lessons were delivered in the format of animated short videos and talk shows |
|  | Who (programme provider): | Bicultural registered dietitian/ health educator |
|  | How (delivery mode and setting): | Delivered using tablet computers |
|  | Where (delivery platform): | Delivered at home-based and technology-mediated |
|  | When and how much: | i) Programme commencement: between 3 and 5 years  ii) How often-programme frequency: 8 weekly lessons  iii) Length of session: 30 minutes (10-15 minutes of animated short videos)  iv) Programme duration: 8 weeks |
|  | Tailoring: | In this intervention, images, food items, and sample menus were used that familiar to the Chinese culture, and easy family physical activities were provided specific to inner cities where the participants resided |
|  | Modifications: | ?*  * Information about the element was not reported/ not sufficiently reported |
|  | How well: | ?*  * Information about the element was not reported/ not sufficiently reported |
