# Supplementary material for: Interventions for positive parental feeding practices in low-income households: a scoping review
Source: Int J Behav Nutr Phys Act. 2026 Jun 17;23:83. doi: 10.1186/s12966-026-01938-5 (PMC13543349; doi:10.1186/s12966-026-01938-5)
Supplement: Supplementary file 2 [file 12966_2026_1938_MOESM2_ESM.docx]

**Table 4** Intervention domains and implementation strategies (n=15)

| No. | Author  (Year) | Targeted Construct | Implementation | Outcome Finding(s) |
| --- | --- | --- | --- | --- |
| A) Parenting-focused domain: Targeted parental feeding style, responsive feeding, scaffolding and interaction quality (7 studies, 46.67%) | | | | |
| 1 | Miller et al., 2025 [21] | Targeted i) responsive feeding, ii) feeding styles, iii) feeding environment | The Smart Beginnings (SB) intervention involved an interventionist to i) videotaped mother-infant interactions during clinic visit, ii) reviewed video clips with mothers and highlighted moments of responsive interaction to reinforce sensitivity to infant cues, iii) guide mothers to practice responsive verbal interaction (talking, reading, and playing) with infants | i) Parental feeding outcomes  -decreased pressuring feeding styles  -increased responsive feeding indirectly via improved cognitive stimulation  -decreased indulgent feeding among intervention group  ii) Child eating/ dietary outcomes  -not primary outcome  iii) Anthropometric outcomes  -not reported  iv) other outcomes  -increased cognitive stimulation  -improved parent-infant interaction quality |
| 2 | Nix et al., 2024 [24] | Targeted i) autonomy support, ii) meal structure, iii) responsive feeding, iv) feeding environment | The Recipe 4 Success intervention (Scaled-up study) involved home visitors to i) observe parent-toddler food preparation, ii) coach in real time, to respond to toddlers’ feeding cues, iii) guide preparation of simple foods, iv) modelling supportive language during feeding, v) provide feedback on parents during practicing scaffolding strategies | i) Parental feeding outcomes  -increased responsive food parenting  -increased autonomy-supportive feeding practices  ii) Child eating/ dietary outcomes  -increased toddler self-regulation/ eating behaviours  -increased healthy eating habits  iii) Anthropometric outcomes  -not reported  iv) other outcomes  -improved parent scaffolding skills |
| 3 | Ling et al., 2024 [25] | Targeted i) meal structure, ii) feeding environment, iii) responsive feeding | The Happy Family, Healthy Kids Program involved parents to i) attend weekly sessions on nutrition and stress management (emotional eating), ii) learn and practice problem-focused coping strategies such as meal planning under stress (creating a supportive home eating environment), iii) participate in cooking activities with their child to reinforced shared meals and routine, iv) complete home practice activities related to mindful eating strategies | i) Parental feeding outcomes  -increased parental feeding knowledge  - increased self-efficacy  -improved feeding environment  ii) Child eating/ dietary outcomes  -increased fruit and vegetable intake  iii) Anthropometric outcomes  -not reported  iv) other outcomes  -decreased food insecurity  -increase coping with stress |
| 4 | Nix et al., 2021 [32] | Targeted i) responsive feeding, ii) autonomy support, iii) feeding styles, iv) feeding environment | A structured food preparation lessons, Recipe 4 Success (Initial study) that focus on eating habits/ self-regulation helped parents to i) engaged in guided food preparation activities, ii) practiced responsive encouragement, iii) practiced non-coercive language and sensitive scaffolding during meals. Trained home visitors delivered the lessons using a manualised curriculum. | i) Parental feeding outcomes  - Increased responsive feeding practices  - Indirectly decreased controlling feeding behaviours  ii) Child eating/ dietary outcomes  - Increased toddler eating habits  iii) Anthropometric outcomes  -not reported  iv) other outcomes  -improved parent confidence |
| 5 | Hughes et al., 2021 [30] and Hughes et al., 2020 [31] | Targeted i) responsive feeding, ii) autonomy support, iii) feeding styles, iv) meal structure | The SEEDS programme involved facilitators to conduct the activities to i) help mothers pay attention to children’s internal cues of hunger and fullness, ii) expose mothers on the challenges while feeding their children through video-based sessions, iii) engaged mothers in experiential learning activities to reinforce the messages in the video-based sessions and guided discussion. | i) Parental feeding outcomes  - Increased responsive and authoritative feeding practices  - Decreased pressuring and parent-centred feeding  ii) Child eating/ dietary outcomes  -not primary outcome  iii) Anthropometric outcomes  -no significant BMI changes  iv) other outcomes  - Increased feeding knowledge and self-efficacy |
| 6 | Messito et al., 2020 [33] | Targeted i) responsive feeding, ii) feeding styles, iii) meal structure | The Starting Early Program (StEP) intervention delivered by trained health educators which involved counseling and support groups through handouts and DVDs to i) help mothers recognising hunger and satiety cues, ii) guide discourage pressuring and restrictive feeding, iii) educate on age-appropriate feeding routines and repeated food exposure, iv) emphasis on responsive bottle-feeding and spoon-feeding. | i) Parental feeding outcomes  - Increased responsive feeding practices at 10 months  - Decreased pressuring feeding behaviours  ii) Child eating/ dietary outcomes  -not primary outcome  iii) Anthropometric outcomes  -not reported  iv) other outcomes  -improved feeding routines |
| 7 | Fisher et al., 2019 [34] | Targeted i) responsive feeding, ii) autonomy support, iii) feeding styles, iv) feeding environment, v) meal structure | The Food, Fun, and Families (FFF) authoritative intervention used graduate-level interventionists to facilitate group sessions, demonstrate and give handouts to help mothers i) emphasis on authoritative feeding by establishing structure and autonomy support in feeding, ii) establishing family eating environment, iii) practise guided choices (parent selects options, child chooses), iv) respons to hunger or satiety cues. | i) Parental feeding outcomes  - Greater number of authoritative parenting practices  ii) Child eating/ dietary outcomes  - Decreased child intake of solid fat and added sugar  iii) Anthropometric outcomes  - No short-term change in child BMI  iv) other outcomes  -improved home eating environment |
|  |  |  |  |  |
| B) Home-based nutrition education and skill-building domain: Emphasized complementary feeding, practical skills, and day-to-day food routines (1 study, 6.67%) | | | | |
| 8 | Gebresadik et al., 2025 [22] | Targeted i) meal structure, ii) responsive feeding | Women Development Army (WDA) used the adapted modules to i) conduct household visits, ii) demonstrate preparation of complementary foods, iii) explain meal frequency and portion sizes, and food consistency iv) conduct group discussion among mothers on feeding challenges, v) reinforce messages during repeated follow-up visits, vi) emphasis on responsive feeding during feeding interactions (recognising hunger/ satiety cues) | i) Parental feeding outcomes  - Increased maternal feeding knowledge  - Increased optimal complementary feeding practices  ii) Child eating/ dietary outcomes  - Increased dietary diversity and meal frequency  iii) Anthropometric outcomes  -not reported  iv) other outcomes  - Alignment with age-appropriate feeding recommendations |
| C) Digital domain: Used mobile phones, apps, SMS, social media, or telehealth to support parental feeding (4 studies, 26.67%) | | | | |
| 9 | Sosanya et al., 2025 [23] | Targeted i) responsive feeding, ii) autonomy support | The BabyThrive mobile application contained i) interactive game modules, ii) quizzes to support parental feeding by immediately feedback on feeding decisions, iii) In-app scenarios reinforced non-coercive feeding responses. Monthly reminder phone calls were made by research staff to encourage app use | i) Parental feeding outcomes  - Increased knowledge on responsive feeding  ii) Child eating/ dietary outcomes  - Increased complementary feeding knowledge and dietary diversity  iii) Anthropometric outcomes  -not reported  iv) other outcomes  -increased engagement |
| 10 | Wen et al., 2023 [28] | Targeted i) meal structure, ii) responsive feeding, iii) feeding environment | A nurse-led telephone-based intervention, mailed intervention booklets and two-way SMS messages provide i) mothers to receive coaching to establish structured family meals, ii) explicit discouragement of eating meals in front of television, iii) guidance on healthy food choices and limiting fast food, iv) support for non-restrictive guidance rather than food rules | i) Parental feeding outcomes  -improved feeding routines  -decreased screen use during meals  ii) Child eating/ dietary outcomes  - Increased meeting dietary behaviour recommendations  iii) Anthropometric outcomes  -not reported  iv) other outcomes  -improved home food practices |
| 11 | Fiks et al., 2017 [35] | Targeted i) responsive feeding, ii) feeding styles, iii) feeding environment | The Grow2Gether, a private social media (Facebook) peer group intervention facilitated by psychologists using curriculum of weekly videos, written posts, and PDFs to i) model responsive infant feeding (recognising hunger, fullness cues, avoiding feeding to soothe), ii) reflect on feeding challenges from peer discussion, iii) provide evidence-based guidance correcting misconceptions, iv) emphasis on non-pressuring, non-restrictive feeding from infancy. | i) Parental feeding outcomes  - Decreased use of food to soothe  -increased responsive feeding beliefs  ii) Child eating/ dietary outcomes  -not primary outcome  iii) Anthropometric outcomes  -No significant difference in infant weight outcome  iv) other outcomes  -peer support  -acceptability |
| 12 | Sun et al., 2017 [36] | Targeted i) responsive feeding, ii) feeding styles, iii) feeding environment | Tablet-based educational modules intervention combining animated short video and talk show format aimed to i) educate on healthy child feeding and portioning, ii) guide on reducing pressure and restriction during meals, iii) educate on appropriate responses to child hunger cues, iv) improve home food stimulus environment. | i) Parental feeding outcomes  - Decreased restrictive feeding practices  - Decreased pressuring feeding behaviours  -Increased maternal self-efficacy for healthy feeding  ii) Child eating/ dietary outcomes  -not primary outcome  iii) Anthropometric outcomes  -no short-term BMI changes  iv) other outcomes  - Improved home food environment |
| D) Multicomponent domain: Integrate parenting-focused, nutrition education and skill-building and digital component (3 studies, 20%) | | | | |
| 13 | Lee et al., 2023 [26] | Targeted i) feeding styles, ii) autonomy support, iii) responsive feeding, iv) feeding environment | An eHealth digital platform which is accessible via smartphone or computer was used to i) teach parents about recognition of child hunger and satiety cues through online modules, ii) provide interactive lessons that discouraged prressuring and restrictive feeding. The provision of biweekly fresh fruits/vegetables/cooking utensils enabled parents i) offering repeated exposure to healthy foods without coercion, ii) to provide guidance on appropriate portion sizes and parent modeling of healthy eating. Parents also received reminder text messages. | i) Parental feeding outcomes  - Increased responsive feeding practices  - Decreased controlling feeding behaviours  ii) Child eating/ dietary outcomes  -not primary outcome  iii) Anthropometric outcomes  -not reported  iv) other outcomes  - Improved parent reported feeding environment |
| 14 | Beck et al., 2023 [27] | Targeted i) autonomy support, ii) responsive feeding | The *Futuros Fuertes* intervention involved face to face sessions with lay health educator and phone-based delivery (SMS reinforcement) conducted to i) coach parents to identify infant hunger and satiety signals, ii) guide parents on not feed to soothe and use alternative calming strategies, iii) promote self-feeding (finger foods, letting infant control intake), iv) introduce parents to practice non-pressuring language during feeding | i) Parental feeding outcomes  - Parent reported shifts towards responsive feeding and less coercive practices  ii) Child eating/ dietary outcomes  - Increased fruit intake at 15 months  iii) Anthropometric outcomes  -not reported  iv) other outcomes  -improved parent confidence |
| 15 | Tovar et al., 2023 [29] | Targeted i) responsive feeding, ii) meal structure, iii) feeding environment | The Strong Families Start at Home intervention involved home visits by community health worker, phone calls, in-home cooking and reading activities, feedback on recorded video, text messages and tailored printed material provided to the parents to i) enhance child diet and feeding routines, ii) set feeding-related goals focusing on regular meals and healthy food availability, iii) promote non-pressuring encouragement of healthy foods, iv) improve home food environment | i) Parental feeding outcomes  - Improvement in structured meal practices  - Increased non-pressing encouragement  ii) Child eating/ dietary outcomes  - Increased diet quality of children  iii) Anthropometric outcomes  -not reported  iv) other outcomes  -improved home food environment |
